# Ecological Risk Assessment and Influencing Factors of Heavy-Metal Leaching From Coal-Based Solid Waste Fly Ash

**DOI:** 10.3389/fchem.2022.932133

**Published:** 2022-07-22

**Authors:** Zhiguo Zhang, Weiqing Cai, Youbiao Hu, Ke Yang, Yonghong Zheng, Chao Fang, Chengnan Ma, Yuning Tan

**Affiliations:** ^1^ School of Earth and Environment, Anhui University of Science and Technology, Huainan, China; ^2^ Institute of Energy, Hefei Comprehensive National Science Center, Hefei, China; ^3^ State Key Laboratory of Mining Response and Disaster Prevention and Control in Deep Coal Mines, Anhui University of Science and Technology, Huainan, China; ^4^ National Engineering Laboratory for Protection of Colliery Eco-environment, Huainan, China

**Keywords:** coal-based solid waste, fly ash, heavy metals, leaching content, risk assessment

## Abstract

In order to promote and broaden the utilization of fly ash as a resource, the fly ash from a 2,660-MW coal-fired power plant in Huainan (China) was investigated. The physical and chemical properties of fly ash were characterized by scanning electron microscopy, energy spectrum analysis, and XRD. The content and different forms of the heavy metals Cd, Cr, Cu, Co, and Ni were determined by acid digestion, oscillation leaching, and Tessier five-step extraction. The effect of pH, temperature, and particle size on the leached amount of heavy metals was studied. Finally, the ecological risk index was calculated for each heavy metal *via* the risk assessment coding (RAC) method and Hakanson ecological risk assessment method, allowing the ecological risk of fly ash to be determined under different environmental conditions. Results showed that the average concentrations of Cd, Cr, Co, and Ni were all below the risk screening values reported for environmental pollutants (pH > 7.5). Under varying pH, temperature, and particle size conditions, the leached amounts (oscillation leaching) were below the soil risk screening values for agricultural land in China. An RAC-Cd value of >50% indicates a high ecological risk, while the RAC values of Co and Ni were between 10 and 30%, indicating a medium ecological risk, and the RAC values of Cr and Cu were <10%, indicating a low ecological risk. With increasing pH, the potential ecological risk index (RI) decreased, with a maximum RI of 59.62 observed at pH 2.8. With increasing temperature, the potential ecological RI increased initially to a maximum of 27.69 at 25°C and then decreased thereafter. With increasing particle size, the ecological RI decreased, with the highest RI of 4.06 occurring at <0.075 mm. The Hakanson ecological RI value was below 150, indicating a slight ecological risk. Therefore, fly ash can be considered as a soil additive and conditioner that is suitable for use in the improvement of reclamation soil in coal mining subsidence areas.

## 1 Introduction

China is rich in coal resources and is one of the major coal-producing countries worldwide. Coal is currently the main energy source used in China (Bai et al., 2018; Xie et al., 2019), reportedly accounting for 56.8% of the total energy consumption in China in 2020 (National Bureau of Statistics, 2020). The 2020 Annual Report on the Development of China’s Coal Industry states that coal production in China increased from 620 million tons in 1978 to 3.41 billion tons in 2016 and 3.90 billion tons in 2020 (China Coal Industry Association, 2020). It has been estimated that before 2050, the proportion of coal in China’s energy structure will remain at about 50%, as the coal-dominated energy structure is difficult to change and coal-based energy is an inevitable choice for national development (Teng et al., 2016; Yuan, 2020). In the process of coal utilization, thermal power generation has the most direct and largest environmental impact (Sahu et al., 2014), with fly ash being a major waste product generated by the combustion of coal in thermal power plants (Nayak et al., 2015).

Fly ash is a non-uniform complex of various minerals, dominated by spherical particles which are mainly composed of sodium, potassium, and calcium aluminum silicate compounds, while also being rich in certain essential elements (such as iron, zinc, manganese, boron, and molybdenum) and toxic elements (such as nickel, chromium, lead, aluminum, and silicon) (Mtarfi et al., 2017; Pandey and Bhattacharya, 2019). At present, the disposal of fly ash by most coal-fired power plants is based on the open-air stacking process, in which fly ash is naturally weathered by the surrounding environment. This process results in the formation of atmospheric dust, which causes air pollution and results in fly ash particles being easily inhaled, presenting a major risk to human health (Liu et al., 2015). In addition, during the open-air stacking process, various substances contained in fly ash dissolve and leach into the surrounding soil and water bodies due to the action of natural factors such as rain, affecting the pH and polluting soils and water bodies (Kostova et al., 2016).

Several studies evaluated the risk of heavy metals by the total concentration of heavy metals in fly ash, but the conclusions were usually inconclusive (Feng et al., 2022; Wang et al., 2022). The characterization of total heavy-metal concentrations in fly ash may indicate elemental enrichment but could not be a good predictor of the availability and toxic behavior of heavy metals in the environment (Pan et al., 2013). In addition, the environmental risk of heavy metals was closely related to their chemical forms, with the soluble and exchangeable fractions of heavy metals also being highly mobile and the residual fractions relatively stable under weathering conditions, rather than being closely related to the total concentration of heavy metals in fly ash (Cai et al., 2019; Liu et al., 2021a). The effective state of heavy metals (water-soluble and exchangeable fractions) under natural conditions is the part that could be easily absorbed and utilized by plants, which can effectively respond to the biological and environmental hazards of heavy metals (Zhang et al., 2022). At present, the mineral phase fugitive morphology of heavy metals in fly ash is mainly based on soil heavy-metal morphology research methods, which include the Tessier method, BCR continuous extraction method, and modified BCR continuous extraction method (Liu et al., 2021b). Among them, the Tessier continuous extraction method is widely used to quantify the different chemically bonded morphologies of heavy metals in sediments. The continuous extraction of fly ash is based on the reaction of fly ash with a sequence of extraction reagents, which contributes to the release of metals from the binding sites (Tessier et al., 1979). Meanwhile, the concentration of leaching of trace heavy-metal elements in fly ash was related to external factors such as pH, temperature, and its own particle size. pH has a direct impact on the solubility of elements and compounds in fly ash. Zhang et al. (2016) studied the effects of different pH conditions on the trace element concentrations in solid waste fly ash effluent, finding that most of the measured heavy metals (such as Cu, Mn, Zn, As, Ag, Cr, Cd, and Pb) followed a cationic leaching pattern, with the concentration of trace elements in the effluent decreasing with increasing fly ash pH. Temperature is also an important factor affecting the leaching of trace elements. Yu et al. (2007) showed that under increasing temperature conditions, Cr dissolution from various types of fly ash increased. However, when the temperature was increased to >40°C, the dissolution of Cr from fly ash stabilized, with no further increase in leaching observed. The size of fly ash particles has also been shown to affect the leaching of elements. Zhou et al. (2015a) investigated the content and morphology of heavy metals in fly ash composed of different particle sizes, finding that volatile metals (such as Zn, Pb, Cu, and Cd) tended to be more abundant in finer particles, resulting in a higher risk to human and environmental health. Furthermore, Tang et al. (2021) also found that heavy metals are more concentrated in finer particulate matter, with the potential ecological risk increasing as the particle size of fly ash decreases. Risk assessment is an important way to evaluate the environmental safety of heavy-metal risks. The current evaluation methods of heavy metals include the single factor index method, the Nemero integrated pollution index method, the ground accumulation index method, the Hakanson ecological risk evaluation method, and the risk assessment code method (Li et al., 2021; Wang et al., 2022; Xu et al., 2022). Among them, the Hakanson ecological risk evaluation method and the risk assessment code method were widely used to evaluate the possible hazards of heavy metals to the environment. Qian et al. (2016) used the Nemerow index to evaluate heavy metals in soil. Furthermore, Tang et al. (2021) used the ecological risk index (RI) to evaluate the risk level of heavy-metal pollution.

At present, fly ash is commonly utilized as an additive to improve soil, with researchers having investigated the physical and chemical properties of the soil before and after treatment, as well as the effect on nutrient indices and beneficial trace element concentrations. However, few studies have investigated the influence of heavy-metal species distribution and abundance or the effect of fly ash's physical and chemical properties on heavy-metal leaching and the subsequent human and ecological risk under varying environmental conditions. In this study, the total amount and morphology of different heavy metals in the ash silo of a coal-fired power plant in Huainan, China, were determined using acid digestion and the Tessier extraction method. The ecological risks posed by heavy metals in fly ash were evaluated using the risk assessment coding (RAC) method and the Hakanson ecological risk index. The aim of this study was to provide scientific guidance and a theoretical basis for the application of fly ash in the Lianghuai mining area of Anhui Province, China, for the improvement of reclaimed soil quality in a manner that ensures the safety of both human and environmental health.

## 2 Materials and Methods

### 2.1 Sample Collection and Pretreatment

Fly ash samples were collected from a large thermal generator in Panji District, Huainan City (Anhui Province, China) with an installed capacity of 2,660 MW, and the annual emission of fly ash is 1.8 million tons. The power plant was a typical pit-mouth power station operated using the “coal-to-electricity integration” model, utilizing coal that mainly originates from the Dingji coal mine, which was constructed at the same time as the power plant. The type of coal used for thermal power generation was mainly gas coal and coking coal.

Once the collected samples were returned to the laboratory, they were dried at 105°C for 24 h and filtered through 200 and 60 mesh sieves to isolate fly ash with particle sizes of <0.075 mm and 0.075–0.25 mm. The different particle size fractions were then stored in sealed bags and labeled for later use.

### 2.2 Fly Ash Physicochemical Analysis

The N, P, K, and organic matter indicators were measured using the Bao (2000). Fly ash morphological observations and compositional analysis were performed using a scanning electron microscope (FlexSEM 1000, Hitachi) and an energy dispersive X-ray fluorescence spectrometer (IXRF 550i, Hitachi), respectively, while the phase composition of fly ash was determined by X-ray diffraction (XRD-6000, Shimadzu). The available heavy metals were extracted from fly ash with DTPA extractant using a method based on the standard protocol, as described previously (HJ 804–2016), while the Cd, Cr, Cu, Co, and Ni concentrations were measured using an inductively coupled plasma mass spectrometer (ICP-MS) (PE NexION 300X, Perkin Elmer). The total content of heavy metals was determined by treating 0.2 g samples with an HNO_3_/HClO_4_/HF acid mixture at 120°C until digestion was complete and the solution was clear. A modified Tessier five-step sequential extraction method (see Table 1) was adopted to fractionate heavy metals into an exchangeable fraction (F1), carbonate-bound fraction (F2), iron-manganese (Fe/Mn) oxide-bound fraction (F3), organic matter/sulfide-bound fraction (F4), and residual fraction (F5) (Lu et al., 2019). After each extraction step, fractions were separated by centrifugation at 3,000 rpm for 30 min, with the supernatant filtered through a 0.45 μM membrane. The residual fraction was also digested and analyzed using the same method. Cd, Cr, Cu, Co, and Ni concentrations in the extracts were determined by ICP-MS. Quality assurance and control were performed using reagent blanks, duplicate sample analysis, and comparison to standard reference soil samples. Statistical data analysis was based on one-way ANOVA by SPSS v.26.0, with Origin 2021 used for plotting images.

**TABLE 1:** Composition and content of surface elements of fly ash.

Element	O	Si	Al	C	Fe	K	Ti	Na	Ca
Mass percentage content (Wt)%	40.65	28.96	21.89	3.31	2.46	1.00	0.65	0.60	0.47
Atomic percentage content (At)%	53.16	21.57	16.97	5.77	0.92	0.53	0.28	0.55	0.25

### 2.3 Leaching Behavior

#### 2.3.1 Effect of Different pH Conditions

The fly ash fraction containing particle sizes of <0.075 mm was dried and 20 g was placed in a jar, with two parallel samples prepared in triplicate. Superior grade pure nitric acid and ultrapure water were combined to prepare 100 ml of nitric acid solution at pH 2.8, 3.9, 4.9, 5.7, and 6.8. The mixtures were then shaken for 30 min at 25°C and 180 r/min and filtered through a 0.45 μM microporous membrane for heavy-metal analysis.

### 2.3.2 Effects of Different Temperatures

The fly ash fraction containing particle sizes of <0.075 mm was dried and 20 g was placed in a jar, with two parallel samples prepared in triplicate for each sample. Samples were combined with 100 ml of ultrapure water and shaken for 30 min at 180 r/min at varying temperatures. According to meteorological data for Huainan (China Weather Network, 2021), the average annual temperature in Huainan ranges from 3 to 29°C, and the temperature in summer reaches 33°C. Therefore, using a temperature-controlled oscillator, the treatment temperatures were set to 0, 10, 15, 20, 25, 30, and 35°C, with the temperature being corrected every 10 min during the experimental period to counteract the influence of ambient temperature. After the oscillation period, samples were removed and left to stand, then filtered through a 0.45 μM microporous membrane prior to analysis.

### 2.3.3 Effects of Different Particle Sizes

Dried fly ash samples were screened to separate fractions containing particle sizes of <0.075 mm and 0.075–0.25 mm, with 20 g of each particle size fraction placed in a jar (two parallel samples prepared in triplicate) and mixed with 100 ml of ultrapure water. The mixtures were then shaken for 30 min at 25°C and 180 r/min and filtered through a 0.45 μM microporous membrane for heavy-metal analysis.

### 2.4 Evaluation Methods

#### 2.4.1 Risk Assessment Coding Evaluation Method

The risk assessment coding (RAC) method and the bioavailability coefficient were used to assess the environmental risk for each sample (Wang et al., 2021), using the following calculation formula:

$$K=\frac{E+C}{Q}\times 100\mathrm{\%,}$$ (1)

where K is the bioavailability coefficient; E is the heavy-metal concentration in the exchangeable state; C is the heavy-metal concentration in the carbonate binding state; and Q is the total sum of heavy-metal concentration. When the overall K value is less than 1%, it is considered risk-free; while K values from 1 to 10% indicate a low risk; 11–30%, a medium risk; 31–50%, a high risk; and >50%, a very high risk.

### 2.4.2 Hakanson Ecological Risk Assessment Method

The Hakanson ecological hazard index (*RI*) evaluation method is based on the characteristics of heavy metals and their environmental behavior, considering the content of heavy metals and their ecological and environmental effects, among which the potential ecological hazard index method involves a single pollution coefficient, a heavy-metal toxicity response coefficient, and a single coefficient of potential ecological hazard (Hakanson, 1980), described as follows:

$$E_{r}^{i}=T_{r}^{i}\frac{C_{s}^{i}}{C_{n}^{i}},$$ (2)

$$\mathrm{RI}=\sum E_{f}^{i},$$ (3)

where *C* ^*i*^_*f*_ is the single pollution coefficient; *C* ^*i*^_*s*_ is the measured concentration of the heavy metal in fly ash; *C* ^*i*^_*n*_ is the minimum soil risk screening value for agricultural land in China; *E* ^*i*^_*r*_ is the single coefficient of potential ecological risk; *T* ^*i*^_*r*_ is the toxic response coefficient for a single pollutant; and *E* ^*i*^_*f*_ is the potential ecological risk index.

Hakanson found that the potential toxicity of heavy metals was generally inversely correlated with their abundance while being positively correlated with their rarity. Therefore, according to the *E* ^*i*^_*r*_ and *RI* values, the assessed heavy metals were divided into different levels of potential ecological hazard (Qian et al., 2016). The canonical treatment rating for the toxic response coefficient of heavy-metal contaminants was Ni = Cu = Co = 5, Cr = 2, and Cd = 30 (Xu et al., 2008). Therefore, the hazard levels were divided into different degrees, and the relationships between *E* ^*i*^_*r*_, *RI*, and the degree of pollution are shown in Table 2.

**TABLE 2:** Physicochemical properties of the fly ash.

Parameter	Value
pH	8.60 ± 0.02
Organic matter (g/kg)	15.93 ± 1.94
Available N (mg/kg)	41.68 ± 1.28
Available P (mg/kg)	15.58 ± 0.23
Available K (mg/kg)	107.48 ± 12.78

## 3 Results and Analysis

### 3.1 Physicochemical Properties of Fly Ash

**FIGURE 1:**
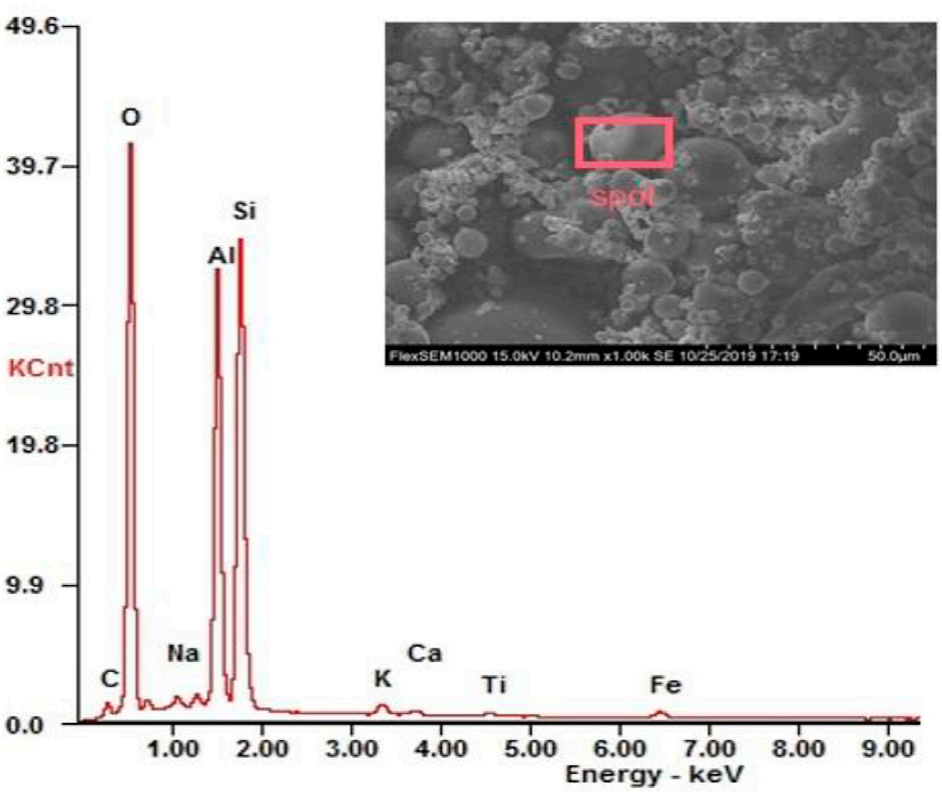
SEM-EDS of fly ash.

As shown in Figure 1, the fly ash samples consisted of spherical microbeads and porous particles of varying particle sizes, with the surface of spherical beads being relatively smooth compared to the porous particles, which generally had an uneven surface, with some pores and cracks. Furthermore, there were numerous small particles attached to the surface of the spherical microbeads, with small diameter particles aggregating to form large particles. As shown in Figure 1 and Table 3, the surface of fly ash contained 9 elements: O, Si, Al, C, Fe, K, Ti, Na, and Ca, among which the concentrations of O, Si, and Al were relatively high, with the total mass fraction and the total atomic fraction of these three elements accounting for 91.5 and 91.7%, respectively. The EDS results did not show the target heavy-metal elements (Cd, Cr, Cu, Co, and Ni), but these heavy metals could be detected by ICP-MS, which is the same as the results of Zhao et al. (2018) and Fu et al. (2019). This may be due to the fact that EDS is a semi-quantitative analysis, which cannot reflect the overall type and content of heavy metals in the sample. The detection limit of SEM-EDS is generally 0.1–0.5%, which is mainly used for point analysis of the main elements of minerals in geochemical mode, and the detection of heavy metals such as Cd, As, and Pb in the measured objects may not reach the detection limit.

**TABLE 3:** Tessier extraction method operation steps.

Steps	Heavy-metal form	Extraction reagents	Extraction conditions
1	Exchangeable fraction (F1)	16 ml 1.0 mol/L Mgcl_2_	Oscillate at 25°C for 1 h
2	Carbonate-bound fraction (F2)	16 ml 1.0 mol/L NaOAc	Oscillate at 25°C for 5 h
3	Fe/Mn oxide-bound fraction (F3)	40 ml 0.04 mol/L NH_3_ClOH	Oscillate at 96°C for 6 h
4	Organic matter/sulfide-bound fraction (F4)	6 ml 0.02 mol/L HN0_3_	85°C water bath for 2 h
		10 ml 30% H_2_O_2_	85°C water bath for 3 h
		6 ml 30% H_2_O_2_	
		10 ml 3.2 mol/L NH_4_OAc	Oscillate at 25°C for 0.5 h
5	Residual fraction (F5)	HNO_3_ + HF + HClO_4_	Dissolve to white or yellowish

**FIGURE 2:**
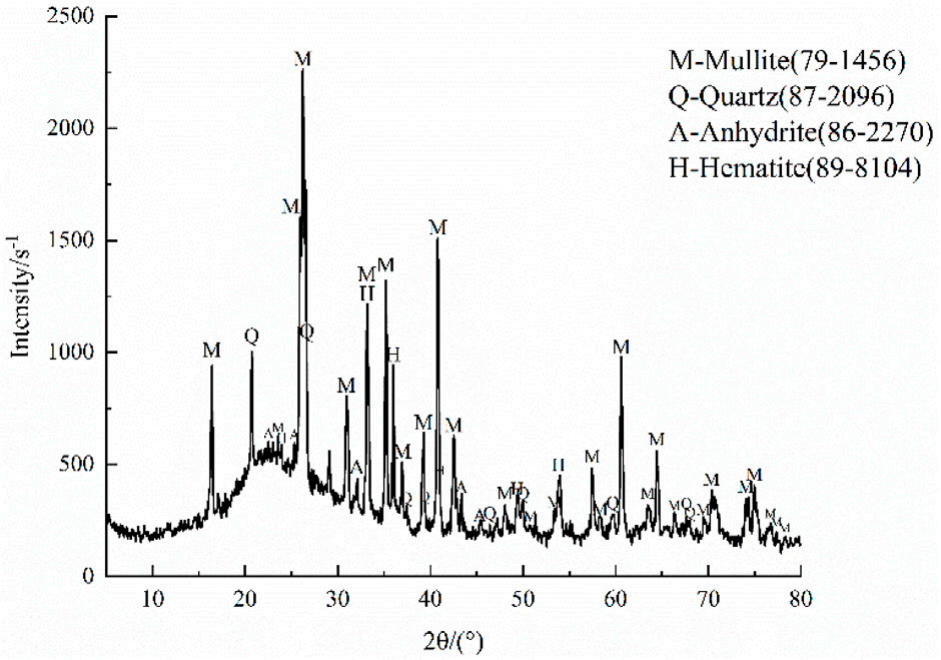
XRD atlas of fly ash.

As shown in Figure 2, the characteristic peaks of fly ash were mainly derived from the crystalline minerals mullite (M) and quartz (Q), with small amounts of hematite (H), anhydrite (A), and other mineral phases. It was established that the mullite content of fly ash was 67.2% and the quartz content was 8.82%, while hematite accounted for 9.0% and anhydrite for 10.0%. According to the nutrient classification standard of the second soil survey in China (China Soil, 1998), the average organic matter content of the fly ash was 15.93 g/kg (Table 4), indicating that the organic matter content was deficient (level four deficiency). The average available nitrogen content was 41.68 mg/kg, corresponding to the maximum deficiency grade of level five. The available phosphorus content was 15.58 mg/kg (medium level), while the average available potassium content of fly ash was high at 107.48 mg/kg (medium level).

**TABLE 4:** Relationship between *E* ^*i*^_*r*_ and *RI* and pollution.

Hazard level	*E* ^*i*^_*r*_	*RI*
Low ecological hazards Ⅰ	*E* ^*i*^_*r*_ < 40	*RI* < 150
Medium ecological hazards Ⅱ	40 ≤ *E* ^*i*^_*r*_ < 80	150 ≤ *RI* < 300
High ecological hazards Ⅲ	80 ≤ *E* ^*i*^_*r*_ < 160	300 ≤ *RI* < 600
High ecological hazards Ⅳ	160 ≤ *E* ^*i*^_*r*_ < 320	
Extremely high ecological hazard Ⅴ	*E* ^*i*^_*r*_ ≥ 320	*RI* ≥ 600

### 3.2 Concentration and Chemical Speciation of Heavy Metals in Fly Ash

A high abundance of heavy metals are attached to the surface of coal during combustion, a large number of which are considered to be toxic and able to be leached from fly ash under atmospheric rainfall or other meteorological conditions, resulting in the pollution of surrounding environments. In this study, the total amount of five harmful heavy-metal elements in different phase fractions was determined, including Cd, Cr, Cu, Co, and Ni.

The heavy-metal content of fly ash is shown in Table 5, which was analyzed according to the Chinese soil environmental quality standard (GB15618-2018, 2018), showing that the average content of Cd, Cr, Cu, and Ni was lower than the environmental pollutant risk screening value (pH > 7.5). Both the total amount and the available content of heavy metals in fly ash exceeded the Huainan soil background value, suggesting that fly ash poses a potential risk of heavy-metal contamination.

**TABLE 5:** Comparison of trace heavy-metal content in fly ash and background value of each element (mg/kg).

Metallic elements		Maximum	Minimum	Average	Standard deviation	Coefficient %	Huainan soil (Wei et al., 2017)	GB15618-2018
Cd	Total	0.38	0.24	0.31	0.1	31.93	0.06	0.6
	Available	0.06	0.03	0.04	0.02	38.57		
Cr	Total	129.5	117.6	123.55	8.41	6.81	64.93	250
	Available	2.2	1.5	1.85	0.49	26.76		
Co	Total	31	30.55	30.78	0.32	1.03	10.74	-
	Available	0.45	0.42	0.44	0.02	4.06		
Ni	Total	62.8	60	61.4	1.98	3.22	25.74	190
	Available	0.38	0.35	0.36	0.02	5.47		
Cu	Total	132.6	126	129.3	4.67	3.61	24.16	100
	Available	2.03	1.77	1.9	0.18	9.62		

**FIGURE 3:**
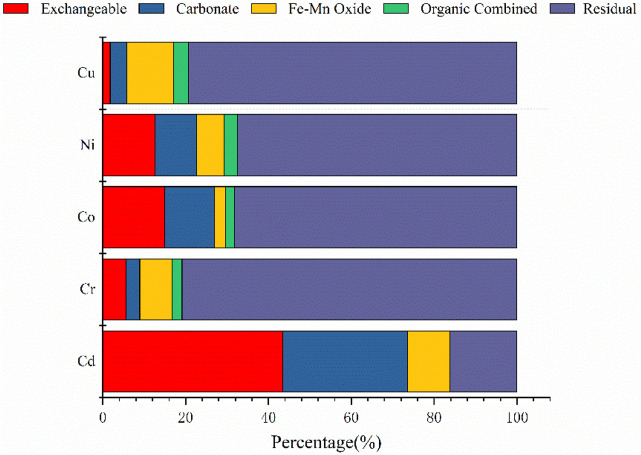
Chemical fractionation of metals in fly ash.

Figure 3 shows the percentage content of different forms of heavy metals in fly ash, with F1 indicating elements in an exchangeable ionic and water-soluble state, F2 indicating elements in a carbonate-bound state, F3 in an iron-manganese oxide state, F4 in an organically bound state, and F5 in a residual state.

As shown in Figure 3, Cd was mainly present in an exchangeable ionic or water-soluble form (43.5%), followed by a carbonate-bound state (30.08%), indicating that a portion of Cd in fly ash migrates easily and presents a high risk of release into the environment. Cr was mainly present in a residual state in fly ash (80.84%), while the content of Cr in the exchangeable state, carbonate-bound state, iron-manganese oxide state, and organic-bound state was 5.63, 3.31, 7.82, and 2.39%, respectively, indicating that Cr is less able to migrate and be converted in the natural environment. Co and Ni were mainly present in fly ash in the residual state, accounting for 68.18 and 67.43%, respectively, followed by the exchangeable state. Therefore, only a small fraction of Co and Ni migrate to the surrounding environment, while Co and Ni in the carbonate-bound state, iron-manganese oxidation state, and organically bound state did not exhibit a significant change, indicating a risk of pollution in the area immediately surrounding the fly ash storage yard. Cu was found to be mainly present in a residual state, an organically bound state, and an iron-manganese oxidation state, with abundances of 79.34, 3.5, and 11.38%, respectively. Cu has a higher boiling point and is entrained in particles with silt during coal combustion, resulting in the Cu mainly existing in a residual state in fly ash. In addition, during the process of weathering, some of the organically bound Cu elements are transferred to the iron-manganese oxide binding state.

Previous research has found that after the addition of fly ash, gangue, and other alkaline substances to the soil, the activity of heavy metals decreases, with their ion-exchange state significantly reduced, while the bound state and residual state of iron and manganese oxides significantly increase, resulting in heavy metals in fly ash being relatively resistant to migration and conversion (Garau et al., 2007; Zhou et al., 2015b). The large specific surface area and alkalinity of fly ash contribute to the precipitation of heavy metals (Cho et al., 2005), with higher ratios of fly ash to soil resulting in higher soil pH levels, increasing the immobilization of heavy metals and significantly decreasing the active state content of heavy metals (Cui et al., 2016).

### 3.3 Main Influencing Factors on Fly Ash Heavy-Metal Leaching

#### 3.3.1 Effect of pH on Fly Ash Heavy-Metal Leaching

**FIGURE 4:**
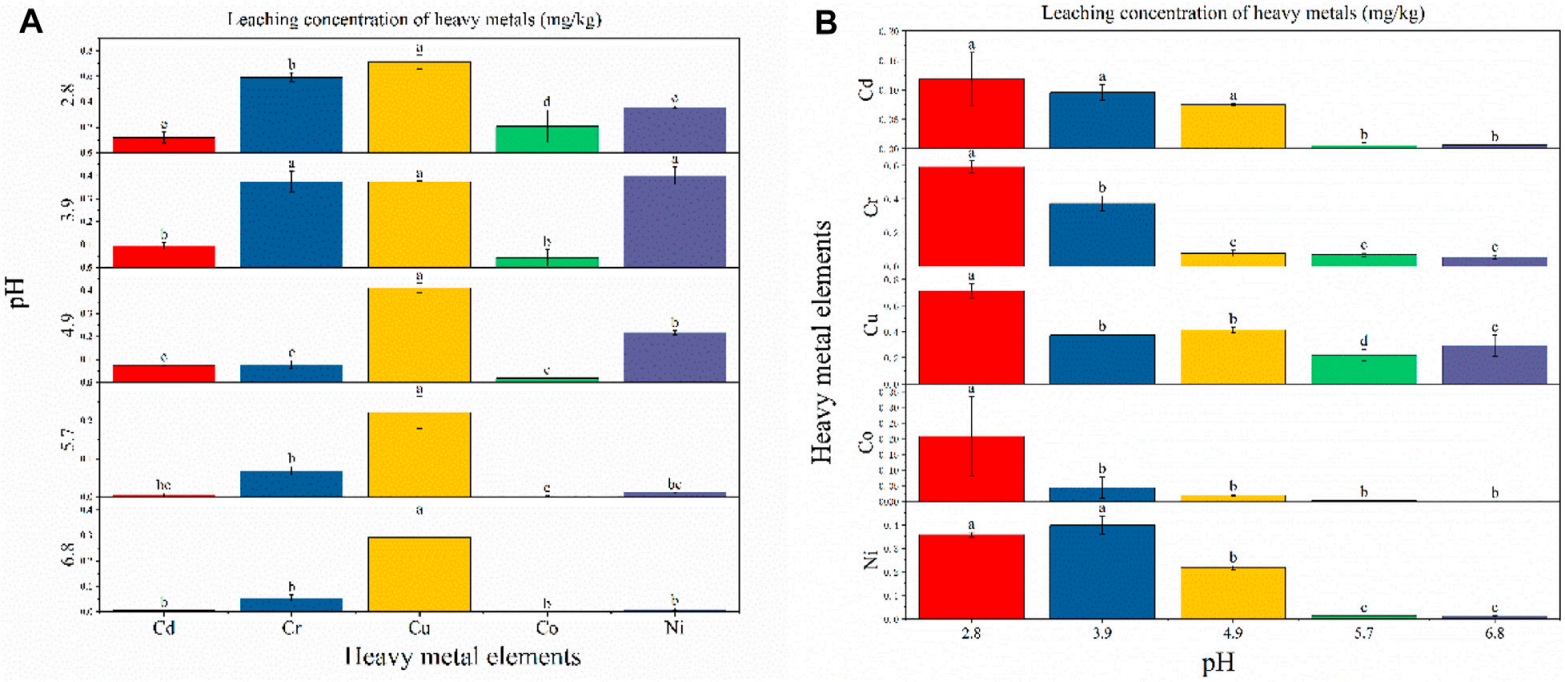
Leaching concentration of heavy metals at different pH conditions: **(A)** difference analysis (Fisher LSD) of different heavy-metal element leaching concentrations under the same pH condition; **(B)** difference analysis of different pH leaching concentrations under the same heavy metal element condition.

Zheng et al. (2022) studies have shown that submersion in different pH level solutions significantly affects the amount of heavy-metal dissolution (Kanokwan et al., 2015; Zheng et al., 2022). Figure 4A shows that when the pH was set at 2.8, 4.9, 5.7, or 6.8, Cu leaching was the highest (0.712 mg/kg, 0.411 mg/kg, 0.221 mg/kg, and 0.291 mg/kg, respectively), while at pH 3.9, Ni exhibited the highest level of leaching (0.399 mg/kg). At pH 2.8, the heavy metals Cd, Cr, Cu, Co, and Ni all exhibited significant differences in their leaching behavior. At pH 3.9, Cu, Cr, and Ni did not exhibit significant differences in their leaching behavior, while they were significantly different from Cd and Co. At pH 4.9, 5.7, and 6.8, the leached amount of Cu was significantly different from that of Cd, Cr, Co, and Ni, while at pH 4.9, Ni leaching differed significantly from Cd, Cr, and Co.

As can be seen from Figure 4B, the leaching of heavy metals shows a gradual trend of reduced leaching as the pH changes from acidic to neutral, which is consistent with the previous findings of Zhang et al. (2016). Most fly ash contains crystal phases of quartz and mullite, with particle surfaces usually covered with a higher abundance of amorphous silicates and a lower abundance of amorphous aluminates (Luo et al., 2017). Liu et al. (2020) reported that heavy metals are generally physically adsorbed on the surface of fly ash particles, while some may also be embedded in the amorphous aluminum silicate component. At a low pH, high H^+^ concentrations destroy the structure of amorphous aluminum silicate and increase heavy-metal ion leaching. With the change in pH from acidic to neutral, the concentration of H^+^ decreased and the amorphous structure gradually stabilized, leading to a decrease in the source of heavy-metal ions and, subsequently, their concentration in the leaching solution. Zhao et al. (2018) found that the leaching solution of fly ash was alkaline, indicating that fly ash leaches alkaline substances and alters the pH of the solution. With the increase in pH, residual alkali components remain in the leaching solution and the generated OH^−^ precipitates with heavy metals, resulting in a decrease in the concentration of heavy-metal ions in the leaching solution.

#### 3.3.2 Effect of Temperature on Fly Ash Heavy-Metal Leaching

**FIGURE 5:**
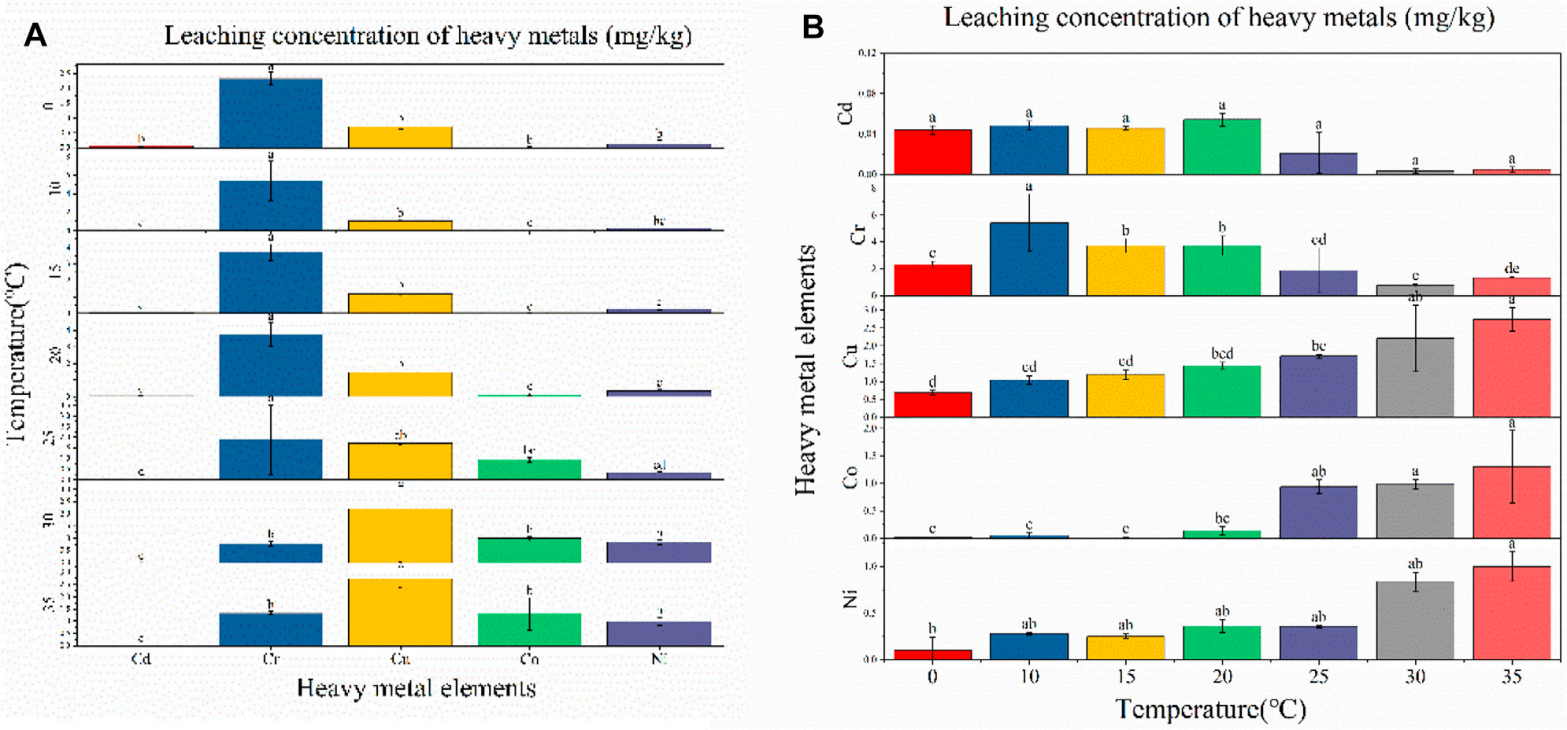
Leaching concentration of heavy metals at different temperature conditions: **(A)** difference analysis of different heavy-metal element leaching concentrations under the same temperature condition; **(B)** difference analysis of different temperature leaching concentrations under the same heavy-metal element condition.

Experiments showed that the temperature of the leaching solution can have a major influence on the amount of heavy-metal leaching. As shown in Figure 5A, at each temperature level, the amount of leaching varied for each heavy metal. At temperatures of 0, 10, 15, 20, and 25°C, Cr exhibited the maximum amount of leaching (2.329 mg/kg, 5.427 mg/kg, 3.708 mg/kg, 3.727 mg/kg, and 1.885 mg/kg, respectively), showing significant differences with Cd, Cu, Co, and Ni at 25°C. At temperatures of 30 and 35°C, Cu leaching was the highest (2.207 mg/kg and 2.739 mg/kg, respectively), exhibiting significant differences compared with Cr, Cd, Co, and Ni.

As shown in Figure 5B, different temperatures did not have significantly different effects on the same heavy metal. Cu, Co, and Ni leaching increased with increasing temperature, which was mainly due to higher temperatures promoting the dissolution and diffusion of heavy metals. This conforms to the Einstein–Stokes equation shown in Eq. 4, where the diffusion coefficient (D) is proportional to the temperature (T), with higher temperatures increasing diffusion.

$$D=\frac{\mathrm{RT}}{6\mathrm{L\pi rn}}.$$ (4)

However, at temperatures of 0, 10, 15, 20, 25, 30, and 35°C, the leached concentration did not change significantly in the present study, exhibiting a downward trend overall. Pan et al. (2013) show a closer correlation between heavy-metal leaching concentrations and the weakly acid-extracted state (exchangeable and carbonate-bound) fractions. Whereas heavy metals in the weakly acid-extracted state fraction were considered to be weakly bound to fly ash (Kirby and Rimstidt, 1993), which represents a potential bioeffectiveness and leaching capacity. The two fractions of Cd accounted for 74.58% (see Figure 3), thus Cd was more easily leached compared to the other four heavy metals, and the total concentration of Cd and the effective concentration of Cd were also less (see table 5), leading to no significant change in leaching when the temperature was varied. The leached concentration of Cr showed a significant downward trend over the tested temperature range. To interpret the leaching pattern of Cr in solid fly ash, two factors were taken into account: 1) the ion type in the leaching solution (e.g., metal ions and acid ions) (Yang et al., 2002; Liu et al., 2015); 2) the leaching experiment is a dynamic process of adsorption and desorption (Gupta et al., 1992). Due to the high total concentration and effective concentration of Cr (Table 5), the rate of adsorption of the heavy metal Cr by fly ash is lower than the rate of desorption at low temperatures, resulting in Cr being released. At high temperatures, the rate of fly ash adsorption of Cr was higher than the desorption rate, increasing the adsorption of Cr adsorbed on the surface of fly ash and, subsequently, reducing its release. It is also possible that the alkaline substances in fly ash are released slowly with increasing temperature, which increases the concentration of Cr precipitate adsorbed on the surface of fly ash and reduces the concentration of Cr in solution.

#### 3.3.3 Effect of Particle Size on Fly Ash Heavy-Metal Leaching

**FIGURE 6:**
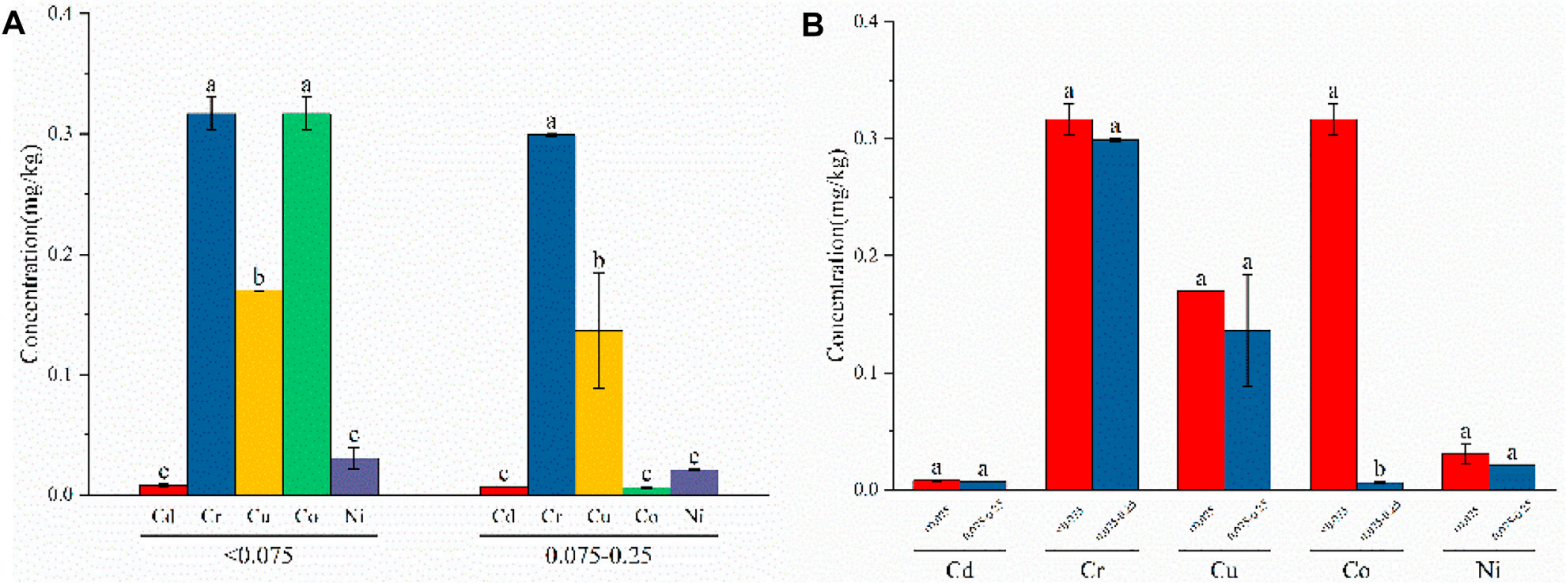
Leaching concentration of heavy metals at different particle sizes conditions: **(A)** difference analysis of different heavy-metal element leaching concentrations under the same particle size conditions; **(B)** difference analysis of different particle size leaching concentrations under the same heavy-metal element condition.

After continuous weathering of fly ash, particles become looser, and the particle size changes accordingly. As shown in Figure 6A, differences were observed in the leaching of heavy metals in each particle size fraction. In the <0.075 mm particle size fraction, the leached concentrations of Cr and Co were 0.317 mg/kg, which were significantly different compared with Cd, Cu, and Ni. The levels of Cd and Ni leaching were minimal and exhibited no significant difference. In the 0.075–0.25 mm particle size fraction, Cr exhibited the largest leaching capacity of 0.299 mg/kg, which was significantly different from the other four metals. Co exhibited the lowest level of leaching at 0.0058 mg/kg, with no significant difference observed compared to Co and Ni, although it was significantly different from Cr and Cu.

As can be seen from Figure 6B, the leaching of heavy metals generally showed an upward trend with a decrease in particle size, which is consistent with the results of Zhou et al. (2015a) and Tang et al. (2021). Cd, Cr, Cu, and Ni leaching increased with decreasing particle size, with no significant differences observed among these metals. The leaching of Co increased with decreasing particle size and was significantly different from the other metals. Small-particle fly ash has a large specific surface area and, therefore, allows more heavy metals to adhere to its surface. Conditions with large particle sizes require sufficient diffusion resistance for heavy metals to leach from within, and the concentration of leachable heavy metals is relatively low.

## 4 Ecological Risk Assessment of Heavy Metals in Fly Ash

### 4.1 Risk Assessment Coding Evaluation Index

**FIGURE 7:**
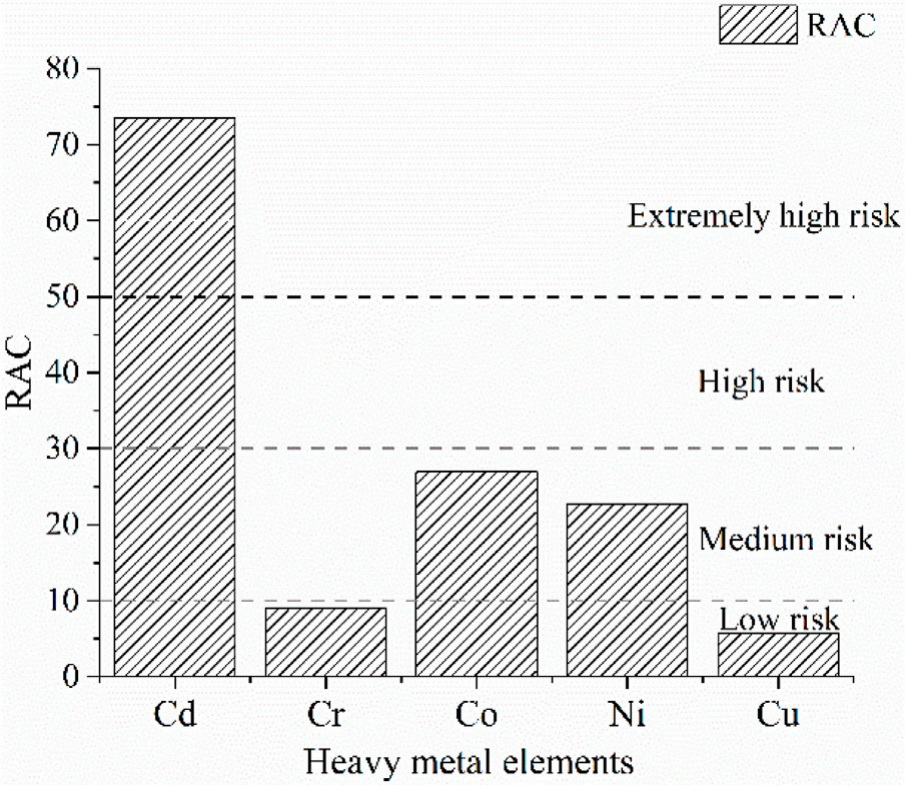
Risk assessment grade of heavy metals.

The RAC evaluation index expresses the ecological risks posed by each form of heavy metal to the environment. As can be seen from Figure 7, RAC-Cd is greater than 50%, indicating a very high ecological risk impact as Cd will easily migrate and be converted under certain environmental conditions. The RAC values of Co and Ni were between 10 and 30%, indicating a medium risk, while the RAC values of Cr and Cu were less than 10%, which indicates a low risk. This reflects the varying risks posed by these five elements in fly ash, depending on their form and phase, which significantly alter their level of hazard to the environment.

### 4.2 The Hakanson Potential Ecological Risk Index Evaluation

#### 4.2.1 Hakanson Potential Ecological Risk Index Under Different pH Conditions

In this study, the potential ecological hazard level of fly ash heavy-metal pollution was evaluated using the Hakanson potential ecological hazard index method, allowing the single potential ecological risk index and potential ecological hazard index to be established for the five heavy-metal elements in fly ash samples under different pH conditions, as shown in Table 6. At pH levels of 2.8, 3.9, 4.9, 5.7, and 6.8, the individual potential risk index values for Cr, Cu, Co, and Ni did not exceed 40, indicating that their pollution levels present a low ecological risk. At pH levels of 4.9, 5.7, and 6.8, Cd pollution levels present a low ecological hazard, while at pH 2.8 and 3.9, a moderate ecological hazard is exhibited. This shows that the heavy-metal element Cd presents the greatest potential ecological risk in fly ash. The potential ecological hazard index *RI* values for the five heavy-metal elements at pH levels of 2.8, 3.9, 4.9, 5.7, and 6.8 were 59.62, 47.96, 37.73, 2.81, and 3.13, respectively, none of which exceeded 150, indicating that they present only a slight ecological hazard. As the pH increases, the potential ecological risk index RI decreases accordingly, with the *RI* being largest at pH 2.8 (59.62). Numerous studies have shown that the soil in the Lianghuai mining area is weakly alkaline (Yang et al., 2019; Sandeep et al., 2016), indicating that fly ash can be safely applied for soil reclamation in the Lianghuai mining area.

**TABLE 6:** Hakanson potential ecological risk index under different pH conditions.

pH	Metals	C_s_^i^	C_n_^i^	E_r_^i^	RI
2.8	Cd	0.1186	0.06	59.28	59.62
	Cr	0.5920	64.93	0.02	
	Cu	0.7117	24.16	0.15	
	Co	0.2071	10.74	0.10	
	Ni	0.3573	25.74	0.07	
3.9	Cd	0.0955	0.06	47.77	47.96
	Cr	0.3737	64.93	0.01	
	Cu	0.3734	24.16	0.08	
	Co	0.0436	10.74	0.02	
	Ni	0.3993	25.74	0.08	
4.9	Cd	0.0752	0.06	37.59	37.73
	Cr	0.0774	64.93	0.00	
	Cu	0.4112	24.16	0.09	
	Co	0.0191	10.74	0.01	
	Ni	0.2175	25.74	0.04	
5.7	Cd	0.0055	0.06	2.76	2.81
	Cr	0.0684	64.93	0.00	
	Cu	0.2211	24.16	0.05	
	Co	0.0029	10.74	0.00	
	Ni	0.0124	25.74	0.00	
6.8	Cd	0.0061	0.06	3.07	3.13
	Cr	0.0539	64.93	0.00	
	Cu	0.2916	24.16	0.06	
	Co	0.0002	10.74	0.00	
	Ni	0.0103	25.74	0.00	

#### 4.2.2 The Hakanson Potential Ecological Risk Index Under Different Temperature Conditions

The potential ecological risk index for the five heavy-metal elements in fly ash samples under different temperature conditions was calculated using the Hakanson formula, as shown in Table 7. At temperatures of 0, 10, 15, 20, 25, 30, and 35°C, the individual potential ecological risk index and the potential ecological risk index for the five heavy metals were all less than 40 and 150, respectively, indicating a low ecological hazard. With increasing temperature, the potential ecological risk index *RI* tended to rise initially and then decline, reaching a maximum *RI* at 25°C (27.69). The Lianghuai region is a typical temperate monsoon region with an average annual temperature of about 15°C (Lu et al., 2017; Xie et al., 2020). At this temperature, the risk of fly ash to the environment is low, further supporting its potential for use as a safe soil conditioner.

**TABLE 7:** Hakanso potential ecological risk index under different temperature conditions.

Temperature (°C)	Metals	C_s_^i^	E_r_^i^	RI
0	Cd	0.0440	22.01	22.25
	Cr	2.3290	0.07	
	Cu	0.6949	0.14	
	Co	0.0237	0.01	
	Ni	0.1030	0.02	
10	Cd	0.0483	24.16	24.62
	Cr	5.4265	0.17	
	Cu	1.0501	0.22	
	Co	0.0527	0.02	
	Ni	0.2813	0.05	
15	Cd	0.0458	22.88	23.30
	Cr	3.7082	0.11	
	Cu	1.1999	0.25	
	Co	0.0121	0.01	
	Ni	0.2531	0.05	
20	Cd	0.0543	27.15	27.69
	Cr	3.7267	0.11	
	Cu	1.4412	0.30	
	Co	0.1385	0.06	
	Ni	0.3623	0.07	
25	Cd	0.0212	10.61	11.54
	Cr	1.8853	0.06	
	Cu	1.7133	0.35	
	Co	0.9635	0.45	
	Ni	0.3572	0.07	
30	Cd	0.0036	1.80	2.90
	Cr	0.7804	0.02	
	Cu	2.2070	0.46	
	Co	0.9969	0.46	
	Ni	0.8382	0.16	
35	Cd	0.0049	2.46	3.90
	Cr	1.3319	0.04	
	Cu	2.7393	0.57	
	Co	1.3781	0.64	
	Ni	0.9995	0.19	

#### 4.2.3 The Hakanson Potential Ecological Risk Index Under Different Particle Size Conditions

The potential ecological risk index for the five heavy-metal elements in fly ash samples under different particle size conditions was calculated using the Hakanson formula, as shown in Table 8.

**TABLE 8:** Hakanson potential ecological risk index of fly ash under different particle size conditions.

Particle (mm)	Metals	C_s_^i^	E_r_^i^	RI
<0.075	Cd	0.0077	3.86	4.06
	Cr	0.3166	0.01	
	Cu	0.1698	0.04	
	Co	0.3166	0.15	
	Ni	0.0305	0.006	
0.25–0.075	Cd	0.0069	3.43	3.48
	Cr	0.2990	0.009	
	Cu	0.1362	0.03	
	Co	0.0058	0.003	
	Ni	0.0208	0.004	

At particle sizes of <0.075 mm and 0.25–0.075 mm, the *E* ^*i*^_r_ of Cd, Cr, Cu, Ni, and Co were less than 40, indicating that the degree of contamination was at the cleaning grade level. The potential ecological hazard index *RI* values for the five heavy-metal elements at particle sizes of <0.075 mm and 0.25–0.075 mm were 4.06 and 3.48, respectively, indicating that fly ash presents a slight ecological hazard. With the increase in particle size, the ecological risk index decreased overall.

## 5 Conclusion

The physicochemical properties, heavy-metal content, morphology, and leached content of heavy metals were determined for fly ash under different pH, temperature, and particle size conditions, with their relative risks established by calculating their ecological risk index and RAC index values. Based on these findings, it can be concluded that 1) the active content and the total amount of Cd, Cr, Cu, and Ni elements in fly ash leaching liquid were less than the corresponding minimum soil risk screening value for agricultural land in China, while the heavy metal Co was not specified in the soil pollution risk control standard for agricultural land. Overall, results indicated that fly ash would have little impact on the soil ecological environment under short-term immersion conditions and, therefore, can be ignored under normal circumstances; 2) generally, a low level of harm is likely to be caused by fly ash to the environment under the three different experimental conditions assessed. In the morphological evaluation (RAC) of heavy metals, Cd exhibited a very high ecological risk, indicating that fly ash may cause an ecological risk to the soil. The long-term accumulation of fly ash would allow the accumulation of trace elements in the soil under leaching conditions, which may also present soil ecological and environmental risks, as well as a risk to public health; 3) under different pH, temperature, and particle size conditions, the oscillating leaching amount of fly ash was less than the soil risk screening value for agricultural land in China. With an increase in pH, the potential ecological risk index *RI* value decreased, with pH 2.8 exhibiting the highest *RI* value of 59.62. Under increasing temperature conditions, the potential ecological risk index RI value tended to rise initially and then decrease, with the highest RI observed at 25°C (27.69). With increasing particle size, the ecological risk index value decreased, exhibiting a maximum *RI* at <0.075 mm of 4.06; 4) fly ash is composed of spherical microbeads and porous particles of different particle sizes, with its microstructure exhibiting good porosity, a high specific surface area, and a strong adsorption capability, with low bulk weight and an abundance of trace elements such as Si, Al, and Fe. Furthermore, the nutrient availability (such as available potassium and available phosphorus) was relatively rich, providing the basic requirements for fly ash as a soil amendment. The results of this study indicate that fly ash can safely be applied to the improvement of reclaimed soil in the Lianghuai mining area, although the potential ecological risks of Cd leaching should be taken into account and monitored. Therefore, the use of environmentally-friendly eluents (chelators and organic acids) should be used to chemically leach fly ash prior to use, reducing the ecological risk of the heavy metal Cd and achieving the safe utilization of fly ash as a sustainable resource.

## Data Availability

The original contributions presented in the study are included in the article/Supplementary Material; further inquiries can be directed to the corresponding author.
